# Optimizing Extended Tight‐Binding Methods for Metal‐Surface Interactions

**DOI:** 10.1002/cphc.202500463

**Published:** 2025-11-12

**Authors:** Siyavash Moradi, Pooria Dabbaghi, Christopher J. Stein

**Affiliations:** ^1^ Department of Chemistry TUM School of Natural Sciences and Catalysis Research Center Technical University of Munich Lichtenbergstr. 4 85748 Garching Germany; ^2^ Department of Energy Conversion and Storage Technical University of Denmark Anker Engelunds Vej 301 2800 Kongens Lyngby Denmark; ^3^ Atomistic Modeling Center Munich Data Science Institute Technical University of Munich Walther‐von‐Dyck Str. 10 85748 Garching Germany

**Keywords:** computational catalysis, parameter optimization, semiempirical methods

## Abstract

The accurate description of metal–water interfaces is essential for understanding processes in heterogeneous catalysis, electrochemistry, and surface science. Capturing the delicate balance between electrostatic and charge‐transfer interactions in these systems, while efficiently sampling configurations to locate minima or approximate thermodynamic ensembles, requires electronic‐structure methods that are both accurate and computationally efficient. Density functional tight‐binding methods have the potential to strike the right balance, and here we demonstrate how systematic parameter optimization within the GFN1‐xTB framework improves the description of water–metal interactions. Using previously published reference data for five metals (Cu, Ag, Au, Pd, Pt) and their (100) and (111) facets, we explore various adsorption sites, orientations, and distances. Sobol sensitivity analysis identifies the most influential parameters for each system, which are then optimized to minimize errors in adsorption energies. This targeted optimization yields substantial accuracy gains, reducing root‐mean‐square errors by approximately 20–60%. The modified method provides reliable predictions for catalytic studies where the default parameterization can fail qualitatively. However, such improvements come at the cost of reduced transferability across systems and properties, emphasizing that parameter optimization must be carefully tailored to the specific chemical context.

## Graphical abstract

This work demonstrates how systematic parameter optimization within GFN1‐xTB (Geometry, Frequency, Noncovalent, eXtended Tight‐Binding) framework improves the accuracy of water–metal interaction energies. Using Sobol sensitivity analysis, key parameters are identified and optimized for copper, silver, gold, palladium, and platinum surfaces. The optimized model achieves significantly lower root‐mean‐square error values.

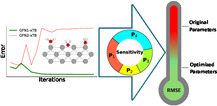

## 1 Introduction

The interaction between water and metal surfaces profoundly influences processes relevant to energy conversion, electrocatalysis, corrosion, and other aspects of surface chemistry at aqueous metal interfaces. While in fuel‐cell technology, water plays a very active role due to water splitting,^[^ ^1^, ^2^ ^]^ in electrochemical CO_2_ reduction, the water layer can facilitate proton transport and stabilize intermediates on transition‐metal electrodes.^[^ ^3^ ^]^ Despite its obvious relevance, the atomistic modeling of water–metal interfaces remains a formidable challenge, as it often requires capturing a delicate interplay of covalent, ionic, and hydrogen‐bonding.^[^ ^4^, ^5^ ^]^ High‐level quantum‐chemical methods such as density functional theory (DFT)^[^ ^6^, ^7^ ^]^ are the method of choice for studying these interfaces, offering accurate insights into preferred adsorption sites, reaction pathways, and energetic barriers.^[^ ^8^, ^9^ ^]^ However, the computational expense can become prohibitive, especially when large supercells, numerous adsorbates, or extended simulation timescales are required in an ab initio molecular dynamics framework.^[^ ^10^ ^]^ These limitations motivate the use of semiempirical approaches such as density functional tight‐binding (DFTB) variants, which come at moderate computational cost while still retaining the essential interactions.^[^ ^11^, ^12^, ^13^ ^]^ Central to these methods is the concept of a minimal valence‐only basis set—frequently a minimal Gaussian‐orbital basis—and the introduction of carefully calibrated parameters to mimic the effect of the underlying interactions in an approximate but computationally efficient manner. Within this family of methods, extended tight‐binding (xTB) has attracted notable attention in recent years, in part due to an element‐specific parameterization as opposed to a pairwise scheme, which renders parameter optimization more feasible and increases the transferability.^[^ ^14^ ^]^ The GFN (Geometries, Frequencies, and Noncovalent interactions) approach, developed by Grimme et al. stands out for its emphasis on capturing a broad range of ground‐state molecular properties for both organic and inorganic molecules and materials.^[^ ^15^, ^16^ ^]^ In this work, we focus on the GFN1 variant due to the empirically observed stable convergence for metallic systems in periodic calculations. It includes a self‐consistent charge (SCC) procedure and distance‐ or coordination‐dependent terms for a realistic description of repulsion, polarization, and dispersion. Several studies have highlighted how reparameterization of DFTB methods can enhance predictive accuracy in a variety of contexts, including solid‐state properties across the periodic table,^[^ ^17^ ^]^ periodic optimizations of metal–organic frameworks,^[^ ^18^ ^]^ multi‐acid–multi‐base atmospheric clusters,^[^ ^19^ ^]^ Th–O systems,^[^ ^20^ ^]^ Au‐nanostructures,^[^ ^21^ ^]^ halide perovskites,^[^ ^22^ ^]^ and the solid‐electrolyte interphase for Li‐ion batteries.^[^ ^23^ ^]^ Regarding optimization methods for DFTB parameterization, automated particle swarm optimization has been demonstrated to enable systematic and transferable parametrization for molecules and solids.^[^ ^24^ ^]^ A semiautomatic Kohn–Sham‐based scheme for homoatomic crystals^[^ ^25^ ^]^ delivered parameters for a large part of the periodic table and was further extended in the QUASINANO2015 parameter set to deliver energies and analytic gradients for elements H–Ca with broadly competitive accuracy.^[^ ^26^ ^]^ Another approach that avoids the need to define element‐pairwise parameters present in several DFTB formulations has been presented by Cui et al.^[^ ^17^ ^]^ who optimize their parameters semiautomatically on a consistent set of artificial homoelemental crystals. Complementing these advances, the DSKO framework constrains electronic Slater–Koster blocks to yield accurate, transferable parameters.^[^ ^27^ ^]^ Data‐driven parameter refinement strategies frequently aim to refit the DFTB repulsive potentials to system‐specific reference data. Unsupervised learning schemes inferring generalized forms from structural descriptors,^[^ ^28^ ^]^ deep‐tensor neural networks capturing many‐body repulsion with ab initio fidelity,^[^ ^29^ ^]^ and the Chebyshev interaction model for efficient simulation (ChIMES)^[^ ^30^ ^]^ are among the methods of choice here. While prior work has showcased encouraging results for molecular crystals and biomolecules,^[^ ^31^, ^32^ ^]^ a systematic validation for the chemically complex water–metal interfaces in periodic calculations is missing for element‐wise parameterized methods such as GFN1‐xTB, but has been published^[^ ^33^ ^]^ for other DFTB variants in combination with a ChIMES correction.^[^ ^34^ ^]^ To harness the efficiency of such an extended tight‐binding scheme for metal–water interfaces, a reparameterization of the method is assumed to greatly enhance the accuracy for this restricted area of chemical space. While DFTB methods often aim at a general and hence transferable parameterization, applications that are restricted to a certain kind of chemistry benefit from a reparameterization or even atom‐typing at the expense of losing transferability. In the current study of water–metal interaction energies, the metals are all modeled as periodic metallic surfaces, whereas in the original xTB parametrizations small transition‐metal complexes—with very different bonding patterns and interaction motifs—were also included in the fitting data.^[^ ^14^ ^]^ In this work, we, therefore, take a systematic route toward refining an extended tight‐binding model for water interacting with several metallic surfaces. We base our parameter optimization on a sensitivity analysis with Sobol indices,^[^ ^35^ ^]^ which quantitatively ranks the impact of each parameter on the interaction energy. Nature‐inspired algorithms such as particle swarm optimization, are valid alternatives if the hyperparameters are adequately chosen, since the main goal of the Sobol analysis here is to reduce the dimensionality of the search space.^[^ ^36^ ^]^ While other frameworks, such as local Bayesian optimization,^[^ ^37^ ^]^ can handle medium‐sized search spaces, they often require extensive hyperparameter tuning and can be computationally expensive. Likewise, advanced global sensitivity methods such as Hilbert–Schmidt independence criterion (HSIC) that incorporate variable selection, help highlight noncritical parameters and further reduce dimensionality.^[^ ^38^ ^]^ Sobol analysis, however, delivers a mathematically rigorous ranking of input effects via its variance‐based decomposition—each subset of variables is assigned an exact share of output variance—whereas HSIC lacks a direct variance‐based interpretability.^[^ ^39^ ^]^ In our previous work,^[^ ^40^ ^]^ we employed Sobol sensitivity analysis to optimize spin‐polarization parameters in the Q‐Chem‐xTB framework.^[^ ^41^ ^]^ This approach allowed us to identify the most influential parameters for a given reference dataset, leading to significant error reduction for specific molecular properties. Concentrating on the most influential parameters then frequently reduces the parameter space to a regime where local optimization methods can be applied. However, we observed limited transferability of optimized parameters across different properties, underscoring the relevance and limitations of property‐specific optimization strategies in computational chemistry. Focusing only on the most relevant parameters aids the stability of the fitting procedure, whereas diverse datasets prevent overfitting. Our training data is taken from a previously published DFT study^[^ ^42^ ^]^ and consists of around 8260 data points for a single water molecule in various orientations with respect to the (100) and (111) facets of pure Cu, Ag, Au, Pd, and Pt. These calculations were performed using Vienna ab initio simulation package^[^ ^8^ ^]^ with the Perdew‐Burke‐Ernzerhof generalized gradient approximation functional,^[^ ^43^ ^]^ the dDsC dispersion correction,^[^ ^44^ ^]^ and a plane‐wave basis set energy cutoff of 400 eV. The Brillouin zone was sampled by 3 × 3 × 1 Monkhorst‐Pack k‐point grid.^[^ ^45^ ^]^ This dataset collectively covers a diverse mix of d‐band fillings and metal–oxygen bond characters. By including multiple surfaces and a variety of adsorption sites, we ensure that the refined parameters capture the essential physics of water adsorption across multiple metallic environments. The optimization procedure proceeds by minimizing the root‐mean‐square error (RMSE) with respect to a set of reference interaction energies, a strategy that parallels prior reparameterization efforts. Importantly, we implement pragmatic bounds on the parameters, guided by both physical reasoning and statistical evidence gleaned from preliminary fits across all metal surfaces. Employing these bounds prevents the parameter search from straying into unphysical regimes while still offering enough leeway for significant improvements in accuracy. By systematically validating the optimized parameter sets against the complete dataset of interaction geometries, we demonstrate that in most cases, relatively minor adjustments to the default parameterization of the extended tight‐binding method yield substantial gains in predictive capability. Our results show that efficiently parameterized single‐particle Hamiltonians combined with carefully tuned problem‐specific parameter optimization enable realistic simulations of metal–water interfaces at a cost far lower than that of conventional DFT. We propose that these optimized parameters, coupled with standard best practices for model verification, will facilitate the study of large‐scale metal–solution systems, such as those relevant to catalysis, corrosion, or electrode processes. The remainder of this article is organized into the following sections. In Section 2, we briefly outline the relevant aspects of the GFN1‐xTB model, with an emphasis on the key parameters whose tuning underlies our approach, and we describe the sensitivity analysis, the reference dataset, and rationale for the bounds that guide the parameter optimization. In Section 3, we present our optimized parameters and examine the resulting error reductions for each metal and facet, highlighting the physical trends that emerge from the reparameterization. We conclude by discussing future directions for systematically refining these methods for broader classes of interfacial phenomena.

## 2 Theory

### 2.1 GFN1‐xTB

GFN1‐xTB is a semiempirical DFTB method that balances computational efficiency and accuracy for large molecular systems. The more recent version, GFN2‐xTB,^[^ ^15^ ^]^ strongly improved the description of electrostatic interactions through inclusion of higher‐order multipole moments. Unfortunately, these improvements make the method more susceptible to convergence issues for metallic clusters or metals in periodic calculations. Therefore, we focus on the optimization of selected parameters within the GFN1‐xTB framework in this contribution. Rather than providing a complete account of the GFN1‐xTB formalism, we describe below some key parameters that influence the method's performance for our specific system of interest: adsorbates on metallic surfaces.

The total GFN1‐xTB energy expression consists of four terms

(1) $$E=E_{\text{el}}+E_{\text{rep}}+E_{\text{disp}}+E_{\text{XB}}$$

where *E*_el_, *E*_rep_, *E*_disp_, and *E*_XB_ represent the electronic, repulsion, dispersion, and halogen‐bonding contributions, respectively. The electronic energy *E*_el_ is the main subject of our optimization efforts and includes contributions from the zero‐order Hamiltonian H_0_, second‐order density fluctuations, and diagonal contributions from the third‐order density fluctuations. These terms depend on several element‐specific parameters, which are included in our sensitivity analysis and optimization and are introduced in the following.

The Slater exponents *ζ* (denoted as *slater* in the sensitivity analysis and parameter tables, vide infra; the notation is chosen according to the GFN1‐xTB parameter file) define the radial decay of the valence‐only slater‐type orbitals (STOs)^[^ ^46^ ^]^ used in the linear combination of atomic orbitals approach

(2) $$\psi_{i}=\sum_{\mu }^{N_{\text{AO}}}c_{\mu i}\phi_{\mu }\left(\zeta ,\text{STO}-mG\right)$$

where $\phi_{\mu }$ are atom‐centered orbitals. These exponents directly affect the overlap integrals in the Hamiltonian matrix elements and hence influence the covalent bonding description.

The coordination‐number‐dependent self‐energy shifts $k_{\text{CN},l}$ and atomic self‐energies $H_{A}^{l}$ (denoted *levels*) are combined to give the effective atomic self‐energy levels $h_{A}^{l}$

(3) $$h_{A}^{l}=H_{A}^{l}\left(1+k_{\text{CN,}l}{\text{CN}}_{A}\right)$$

These shifts modulate the energetic gap between different orbital shells, affecting hybridization and bonding. They are contracted with overlap matrix elements in the calculation of the zeroth‐order Hamiltonian matrix. Only the atomic self‐energies are element‐specific and will be considered in our optimization study.

The third‐order density fluctuation term includes the atomic Hubbard derivative $\Gamma_{A}$ (denoted *gam3*), which describes charge‐dependent corrections

(4) $$\frac{1}{3}\sum_{A}\Gamma_{A}q_{A}^{3}$$

where *q*_A_ is the Mulliken charge of atom A.

The average chemical hardness *η* enters the modified Coulomb‐interaction term and is defined as

(5) $$\eta =2{\left(\frac{1}{\left(1+\kappa_{A}^{l}\right)\eta_{A}}+\frac{1}{\left(1+\kappa_{B}^{l^{\prime }}\right)\eta_{B}}\right)}^{-1}$$

We include the atomic Hubbard parameters *η*_A/B_ (denoted *gam*) as element‐specific but shell‐independent parameters in our system‐specific approach.

The polynomial term $\Pi \left(R_{\text{AB},\text{ll}\prime }\right)$ is a distance‐ and shell‐dependent fine‐tuning to the electronic energy

(6) $$\Pi \left(R_{\text{AB},\text{ll}\prime }\right)=\left(1+k_{\text{A,}l}^{\text{poly}}{\left(\frac{R_{\text{AB}}}{R_{\text{cov,AB}}}\right)}^{1/2}\right)\left(1+k_{\text{B,}l^{\prime }}^{\text{poly}}{\left(\frac{R_{\text{AB}}}{R_{\text{cov,AB}}}\right)}^{1/2}\right)$$

Here, $k_{\text{A/B}}^{\text{poly}}$ (denoted *shpoly*) are the polynomial expansion coefficients and *R*_cov,AB_ is the parameterized covalent reference distance. These coefficients enhance flexibility in describing covalent interactions.

Since the D3 dispersion correction is very accurate also in the context of DFT calculations, and we do not have any halogen bonding in our systems, the only remaining term with element‐specific parameters is the repulsion energy term *E*_erp_, which is modeled using an atom‐pairwise potential

(7) $$E_{\text{rep}}=\sum_{AB}\frac{Z_{A}^{e\text{ff}}Z_{B}^{e\text{ff}}}{R_{\text{AB}}}e^{-{\left(\alpha_{A}\alpha_{B}\right)}^{1/2}R_{\text{AB}}^{k_{f}}}$$

Here, *Z* ^eff^ (denoted *zeff*) are effective nuclear charges that account for screening effects and are fitted to reproduce reference data, and *α* (denoted *arep*) are repulsion exponents that control the steepness of the repulsion term. The global parameter *k*_f_ is not element‐specific and will not be optimized in our approach.

By focusing on these key parameters, our optimization strategy leverages the flexibility of GFN1‐xTB to achieve significant improvements in predictive a ccuracy.

### 2.2 Sensitivity Analysis and Parameter Optimization

We carried out a sensitivity analysis using Sobol indices to identify which parameters most strongly impact the computed interaction energy using the SALib library.^[^ ^47^ ^]^ This method quantifies the relative importance of each parameter by decomposing the variance in the model output (i.e., the interaction energy) across different regions of the parameter space. In all cases, we have calculated the first‐order and total‐order Sobol indices, where the latter incorporates the effect on the output if several parameters are changed simultaneously. More details of the Sobol approach as applied to parameter optimization are available in our prior work.^[^ ^40^ ^]^ By ranking parameters based on their Sobol indices, we ensure that only the most relevant parameters are selected for optimization. This approach not only reduces computational complexity but also focuses on parameters with the greatest potential to improve predictive accuracy thereby reducing the dimensionality of the problem and expediting convergence. The final optimization of GFN1‐xTB parameters is carried out using the Powell algorithm, a gradient‐free optimization method well‐suited for problems with few variables. The objective function minimized during optimization is the RMSE between the predicted and reference interaction energies

(8) $$\text{RMSE}=\sqrt{\frac{1}{N}\sum_{i\;=\;1}^{N}{\left(E_{i}^{\text{pred}}-E_{i}^{\text{ref}}\right)}^{2}}\;$$

Reference energies are taken from a published DFT dataset of water‐surface interaction configurations for multiple metal surfaces (Cu, Ag, Au, Pd, Pt) and two crystal facets (100 and 111).^[^ ^42^ ^]^ Each configuration consists of a single water molecule in various orientations and distances relative to a metallic slab (≈826 configurations per facet), which in turn is constructed as a p(3 × 3) surface unit cell with sufficient vacuum separation to avoid spurious periodic interactions. By sampling different adsorption sites (bridge, top, hollow) and angular orientations (e.g., cartwheel, propeller angles), the set ensures that the optimizations capture a diverse range of local bonding environments. This diversity is crucial for deriving parameters that remain robust across different surface geometries and local coordination scenarios. The sensitivity analysis and subsequent parameter optimization are carried out using the DFTB+ software.^[^ ^48^ ^]^ To ensure convergence to meaningful parameters, we impose bounds during the optimization. These bounds are determined based on several considerations as follows.

#### 2.2.1 Sign and Shell Order Conservation

For all shell‐dependent parameters with a nonzero default value, the sign is held fixed to avoid unphysical values (e.g., negative Slater exponents). Additionally, the shell hierarchy (e.g., $\zeta_{2s}>\zeta_{2p}$ for Slater exponents in oxygen) is strictly maintained.

#### 2.2.2 Use of Existing Ranges

For each parameter, we determine a plausible lower and upper bound by examining the minimum and maximum values observed across the default parameters of all elements. The rationale is that the diversity of the periodic table is reflected in the parameter range. This is a soft criterion and we check in an individual optimization if a result outside this bound strongly improves the result and can be rationalized in light of the results for the other parameters.

#### 2.2.3 Order‐of‐Magnitude Restrictions

If the RMSE cannot be reduced significantly, we relax the bounds to only preserve the order of magnitude of the parameters. For example, if a parameter has a default value of 2, its bounds are set to [1,9.99]. This approach allows sufficient flexibility to improve the fit without risking an extreme or unphysical growth in the parameter value.

Because the optimization is high‐dimensional and each parameter may exhibit widely varying sensitivity or physically meaningful ranges, we need to impose these sets of customized bounds, especially since we are using a local optimization method. Such restrictions prove essential to guiding the search in a complex, multi‐dimensional parameter space, helping the Powell method converge to physically realistic minima and preventing the optimizer from exploring unphysical regions. As can be seen from the results, the final parameters often remain close to their initial guesses, reflecting physically plausible adjustments that lead to significant RMSE reductions.

## 3 Results

We summarize the results of the parameter optimization in two parts. In the first part, we show the results of a system‐specific optimization where each metal facet is separately optimized. This allows us to assess a best‐case scenario for the optimization procedure in terms of a reduction of the RMSE while we expect little transferability from this procedure. In the second part, we first optimize the parameters for water based on reference data from all facets and subsequently optimize the metal parameters individually, keeping the water parameters fixed.

**Figure 1:**
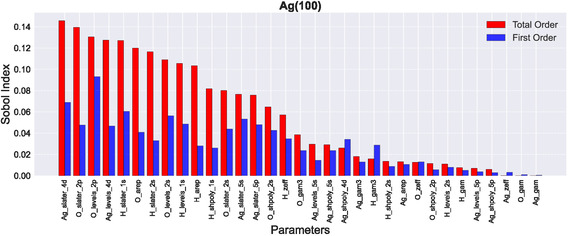
Total‐order (red) and first‐order (blue) Sobol sensitivity indices for Ag(100). Similar plots for the remaining surface facets can be found in the Supporting Information.

**Figure** **1** shows an exemplary bar plot or sensitivity diagram of the calculated Sobol indices for the Ag(100) surface (the remaining plots for all other surfaces are displayed in the Supporting Information). The naming scheme (or key) for the parameters has been introduced in the previous section (levels for $H_{A}^{l}$, slater for *ζ*, gam3 for Γ, gam for *η*, shpoly for $k_{\text{A/B}}^{\text{poly}}$, zeff for *Z*_eff_, and arep for *α*). In the sensitivity diagrams, the first part of the parameter name is the element, the second part is the parameter key, and if the parameter is shell‐dependent, the third part is the shell specification. In general, we consider seven parameter types as introduced above, and since some of them are shell‐dependent, there are 33 parameters in total.

Before we proceed with the discussion of the results obtained from the system‐specific and the more general approach, two comments are in place. First, in the original GFN1‐xTB article, the authors recommend to tune the *K*_AB_ parameter in the Hückel‐type zeroth‐order contribution to yield a system‐specific adaptation of the approach. However, we found that the default value of *K*_AB_ = 1 for the metal‐oxygen pairs resulted in the lowest average errors (see the Supporting Information for more details). Second, we removed four notorious outlier structures for Cu(111).^[^ ^49^, ^50^ ^]^ In initial tests, the element‐specific optimization attempt reduced the RMSE only marginally, from 11.33 to 11.21 kcal mol^−1^, showing a significantly higher initial error than that of Cu(100). Indeed, compared to the other metals, where the difference in RMSE between the (111) and (100) facets never exceeded 2.7 kcal mol^−1^ and optimization yielded more substantial improvements, Cu(111) exhibited both a larger initial discrepancy and very little reduction after optimization. This prompted us to analyze the origin of this high error in more detail, revealing four outlier structures which we then subsequently removed from the dataset to not bias our optimization toward these structures. Although no similar behavior has been observed for the other facets and metals, we carried out the same analysis for the whole dataset to confirm the absence of outliers. The Supporting Information includes bar plots for Cu(111), Cu(100), and Pt(100) showing the 50 structures with the largest errors for both the initial and optimized parameter sets. Notably, only the four data points for Cu(111) continued to exhibit persistently high errors even after optimization.

### 3.1 System‐Specific Optimization

To get an idea of the improvement that can be achieved through reparameterization, we optimized parameters using reference data for each surface facet separately. For this initial test, we decided to optimize only those parameters where the total Sobol index was higher than 0.1. This is a rather arbitrary choice but limits the number of parameters to a number that can still be efficiently optimized with a local optimizer and certainly allows us to assess the potential of a reparameterization. We note that in this system‐specific parameterization, we include parameters from the metals and the adsorbing water (see the Supporting Information for the exact parameters as well as their optimized values).

In **Table** **1**, we compare the RMSEs of the ten individual surface facets obtained with the default parameters and the system‐specific optimizations. In almost all cases, we observe a significant reduction of the RMSE, frequently exceeding 30% improvement. While these results are slightly worse than a nonreactive force‐field optimization of the same dataset^[^ ^42^ ^]^ this is nevertheless a very encouraging result since especially in the challenging cases of Pd(100) and Pt(100) that exhibit a rather large RMSE with the default parameters, our reoptimization brings the method into the realm of qualitatively correct calculations that can be used for explorative studies in catalysis. The fact that the RMSE remains rather large for the Pd(111) facet is a bit surprising at this point, but also certainly due to the fact that for this facet, only three parameters were optimized due to the Sobol sensitivity cutoff chosen. Our alternative optimization schemes discussed below will show that the error can be significantly reduced. As a cross‐check, we repeated the optimization for every facet but fed the parameters to the Powell routine in the exact opposite order of their sensitivity ranking. This reversed‐order run produced a different set of fitted values and an increased RMSE (see the tables in the Supporting Information). The result confirms that in a high‐dimensional space explored with a local method like Powell, the final solution can depend strongly on the order in which parameters are optimized. Our results further demonstrate that the RMSE on (100) facets is generally reduced more effectively than on (111) facets after parameter optimization. This trend likely arises from (100) facets’ higher symmetry and uniform fourfold hollow sites, which simplify electronic structure corrections compared to (111) facets’ heterogeneous threefold face‐centered cubic/ hexagonal close‐packed sites.^[^ ^51^ ^]^ At this point, a short discussion about the physical relevance of the optimized parameters is in place before we discuss alternative and more transferable optimization schemes.

**Table 1:** RMSE (kcal mol^−1^) for the water adsorption dataset across ten metal surface facets for the default GFN1‐xTB parameters and the system‐specific (single‐slab) optimization.

Facet	Default	System specific
Ag(100)	3.34	1.63
Ag(111)	2.81	1.92
Au(100)	3.67	1.83
Au(111)	3.51	2.16
Cu(100)	3.09	1.98
Cu(111)	4.82	4.46
Pd(100)	10.42	5.57
Pd(111)	12.33	10.80
Pt(100)	6.82	3.02
Pt(111)	4.13	2.73

Larger Slater exponents (*ζ*) concentrate the electron density closer to the nucleus (making orbitals more compact), while smaller values lead to more diffuse orbitals. In our optimized parameter sets, certain metals (e.g., Au(100), Au(111)) converge to higher Slater exponents for their p orbitals than the default values. Conversely, the water exponents (e.g., O_slater_2p in Ag(100) and Ag(111)) decrease, indicating more diffuse oxygen 2p orbitals. Such adjustments can mitigate an overestimation of the water–metal bond strength when the adsorbate wavefunction is too localized, thus bringing the computed interaction energies into closer agreement with reference data.

In most of the cases, we observe a strong shift of the metal atomic self‐energies $H_{A}^{l}$ toward lower values, affecting the hybridization. This clearly has a profound effect on the absolute energies but only relative interaction energies are included in our objective function (see Equation 8). More complex objective functions that include other properties like ionization potentials that are strongly correlated with the atomic self‐energies will result in more modest changes of the optimized parameters. We will elaborate on this point in the discussion of the transferability.

Other parameters that were identified as being sensitive to optimization in the Sobol analysis where the repulsion exponents *α* in case of Ag that are slightly increased. Finally, parameters affecting the electrostatic interaction, such as the atomic Hubbard parameters *η* and its charge derivatives Γ, were also among those parameters we optimized. These parameters modify the on‐site energy contributions as a function of deviations from the reference charges. The *η* parameter enters the second‐order SCC term, while Γ appears in the third‐order term. While we observe larger negative values for Γ in oxygen, the picture is more diverse for the other elements and no general physical explanation can be extracted.

### 3.2 General Optimization

In addition to the system‐specific (facet‐by‐facet) optimization, we carried out a more general optimization intended to yield a set of parameters that is more transferable. In fact, since H and O only appear in water in our training set, we optimized those parameters separately, followed by an optimization of the individual metallic elements, but not for each facet separately. Therefore, in the first step, we identify a core subset of water parameters (those associated with oxygen and hydrogen) that consistently appear to be high‐ranked in the Sobol analysis for all metal facets considered. These are somewhat unsurprisingly the eight Slater exponents and atomic levels of both elements.

We only expect rather small changes of the parameters, allowing for a hybridization that is similar to the original parameterization. However, for oxygen, the orbital energy levels approach near‐degeneracy indicating that the parameters obtained here will certainly not be transferable to other observables or molecules containing oxygen. The sensitivity of other parameters that appear at higher order of the DFTB expansion and describe the electrostatic and exchange interaction is more surface and structure dependent and we decided against a reoptimization to avoid overfitting.

We then randomly selected 2000 structures across all surface facets to serve as our training set for the optimization. This subset still ensures broad coverage of relevant interaction geometries. Optimization of these eight parameters lowers the RMSE from 7.80 to 5.91 kcal mol^−1^ for the training set. As evident from the penultimate column in Table 3, the RMSE improves significantly for all surface facets. Notably, for the Pd surface, the RMSE is reduced by ≈30%.

With these water parameters fixed at their optimized values, we next optimize the most sensitive metal parameters individually. As parameters to be optimized, we chose—again somewhat arbitrarily—all parameters with Sobol indices larger than 0.05. If a Slater exponent or an energy levels parameter was selected in this way, we always made sure to include both. The details about the optimization can be found in Table 3. For Ag, the final RMSE drops from about 3.05 only slightly to 2.89 kcal mol^−1^. Notable changes include an increase in the Slater exponents, indicating more diffuse orbitals. In addition, the at times significant changes of the energy levels indicate the vastly different bonding situation in a real, periodic metal slab, compared to the transition‐metal systems used in the original parameterization. In case of the Au surfaces, the final RMSE of 2.41 kcal mol^−1^ is a notable improvement over the initial 3.59 kcal mol^−1^. The increase in $\eta_{\text{Au}}$ also suggests a higher penalty for large charge fluctuations on Au sites. For copper, we achieve an optimized RMSE of 3.34 kcal mol^−1^ which is notably lower than the original 4.14 kcal mol^−1^. Also here, we see that all the levels parameters are reduced. This is also the case for the Pd surfaces, where the optimization leads to a drastic improvement of the RMSE from 11.42 kcal mol^−1^ down to 5.14 kcal mol^−1^. In this case, the optimization really brings the calculation in the range of qualitatively correct results which was not the case before. Finally, for platinum, we observe an RMSE reduction of 45% from 6.22 to 3.43 kcal mol^−1^.

To verify that our procedure has converged to a stable minimum, we repeated the water‐parameter optimization with the optimized metal parameters. The second optimization cycle does not lead to any further improvement—the RMSE is basically unchanged at 5.90 kcal mol^−1^ for the whole dataset—giving us confidence that the parameters are well‐converged. Closer inspection of **Table** **2** shows that during the second iteration of the water parameter optimization, the expected shell hierarchy is violated: the hydrogen Slater exponents approach even closer near‐degeneracy (H_slater_1s≈H_slater_2s), and the atomic self‐energies for oxygen (O_levels_2s≈O_levels_2p) also converge to almost the same value. The shell‐order constraint enforced preserved the physically correct energy separation but without this constraint, the local minimization procedure with the given objective function can interchange shell identities, yielding unphysical orderings. To illustrate the effect of the constraint, we removed the shell‐order constraint and observed a slight decrease in RMSE to 5.73 kcal mol^−1^. However, this came at the cost of four parameter swaps (H_slater_1s with H_slater_2s and O_levels_2s with O_levels_2p) which violates the physical shell order, as discussed above. Because the first‐cycle parameters respect shell order while providing an RMSE that is chemically indistinguishable from the second‐cycle value, we adopt those parameters as an acceptable solution.

**Table 2:** Initial versus first cycle and second cycle optimized water parameters including 2000 randomly selected structures in the optimization.

Parameter	Initial	First cycle optimized	Second cycle optimized
O_slater_2p	2.15306	1.867336	1.855321
H_slater_2s	1.993207	1.854766	1.7960107
H_slater_1s	1.20794	1.766576	1.795441
O_slater_2s	2.345365	2.574092	2.583258
O_levels_2s	−23.398376	−15.952805	−14.98912
O_levels_2p	−17.886554	−14.817483	−14.781212
H_levels_1s	−10.923452	−12.243638	−12.254447
H_levels_2s	−2.171902	−4.316475	−4.40221

The system‐specific approach generally yields the largest reduction in RMSE for each facet, as it is tailored to that particular metal surface. However, the metal‐level (plus optimized water) parameters also provide a substantial improvement over the default values without requiring a fully separate re‐optimization for each new facet. Notably, Pd(111) drops from an RMSE of over 12.33 kcal mol^−1^ under the default parameters to about 10.80 kcal mol^−1^ with a single‐slab fit, and down to 5.14 kcal mol^−1^ under the metal‐level approach. The fact that the system‐specific approach here yields a larger RMSE than the more global scheme indicates that either more parameters would have to be included to significantly reduce the RMSE or that the separate optimization of metal and water parameters avoided the optimization to be stuck in a local minimum. Although the metal‐level RMSEs are slightly higher than those from the system‐specific fits in some cases, the overall reductions confirm that optimized parameters can significantly improve predictions while maintaining transferability within the application domain.

### 3.3 Adsorption and Correlation Plots

To further evaluate the performance of our optimized GFN1‐xTB parameters and as an in‐scope transferability check, we compute interaction energy curves for a single water molecule for an on top site of each metal facet at various distances from the surface. We compare curves obtained with the reference DFT method, and DFTB with the default GFN1‐xTB and the optimized GFN1‐xTB parameters. The reference DFT calculations for the adsorption energy curves are carried out using the same computational setup as the training data.

**Figure 2:**
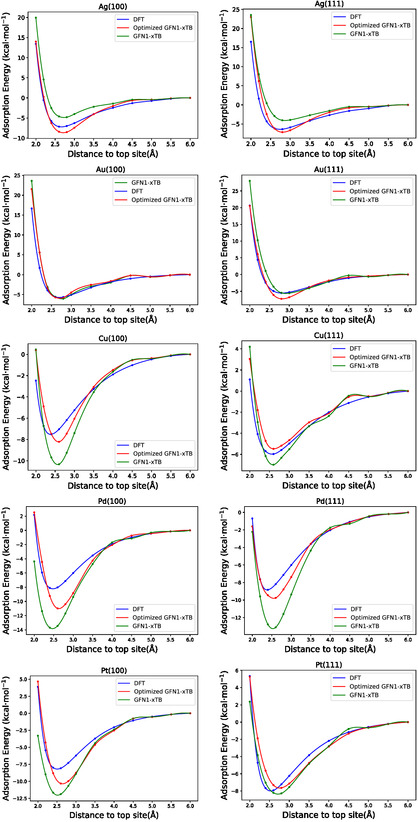
Adsorption energy curves for the atop site of each facet calculated with DFT (blue), and GFN1‐xTB using the default (green) and our optimized (red) parameter set.

**Figure** **2** provides the results for each facet individually. The default GFN1‐xTB results often exhibit an over‐ or underestimation of both the adsorption well‐depth and the equilibrium distance as well as unphysical “wiggles”. These wiggles are an artifact of the method and not of our reparameterization, since they also appear and are even more pronounced for the original parameterization. In fact, especially Au(111), Pd(100), Pd(111), and Pt(111), the curves obtained with our reparameterization are significantly smoother than the original curves. By contrast, the optimized GFN1‐xTB curves generally track the DFT reference more closely, particularly in the near‐equilibrium region. Overall, these adsorption curves confirm that our optimized parameters improve GFN1‐xTB's predictions for water on metal surfaces. In no case is the performance worse than that obtained with default parameters although in case of Pd(100) and Pt(100) the curves still differ considerably from the reference which is also reflected in the RMSEs for all structures.

As a further check for transferability, we evaluated relative energies of H‐up/H‐down bilayers on Pt(111). For this, we constructed a four‐layer($2\times \sqrt{3}$) Pt(111) slab with 16 water molecules in total, following ref. [52]. With our optimized GFN1‐xTB parameters, H‐down is favored over H‐up (−4.09 vs −3.19 eV), whereas the default GFN1‐xTB parameters result in an almost equal relative energy (−2.04 vs −2.14 eV). Our optimized results are now in qualitative agreement with the DFT reference data which also favors the H‐down conformation (−11.17 vs −9.44 eV). This leads to an improved description of the interfacial behavior resulting in a more realistic potential of zero charge and interfacial capacitance. The adsorption strength, however, is still underestimated but greatly improved.

Beyond water, we also tested the default and optimized parameter sets for CO adsorption on these metal surfaces; the corresponding energy curves are provided in the Supporting Information. In these CO adsorption calculations, both the default and the optimized parameters yield considerably larger deviations from DFT, indicating that parameter sets optimized for water do not at all transfer to other adsorbates such as CO. We report this here to highlight that while significant improvements are possible with reoptimizations for very specific problems and observables, they always come at the cost of loss of transferability. If accurate results are desired for CO or any other molecule, a similar strategy of careful data selection, sensitivity analysis, and parameter re‐fitting must be undertaken for all molecules to be considered. Nevertheless, for a given chemical problem, due to the inherent efficiency of GFN1‐xTB a parameter optimization as presented here is doable as an initial step of a new investigation. As a relatively inexpensive semiempirical method, its computational speed permits systematic re‐optimization of parameters with minimal overhead, enabling one to tailor the method to specific adsorbates or reaction scenarios and thereby achieve accuracy improvements that rival those of more computationally demanding electronic‐structure approaches, provided that a representative training set of high‐level reference data is available.

**Figure 3:**
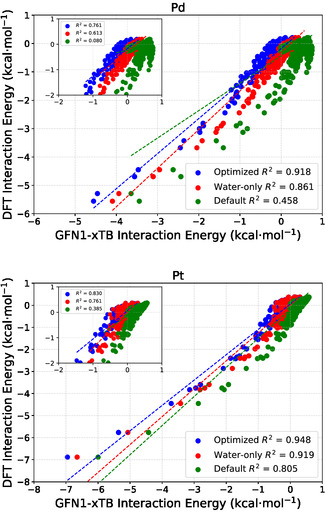
Correlation plots for Pd and Pt comparing three parameter sets with the DFT reference data: optimized (blue), water‐only (red), and default (green). The main plots display the full range of data with corresponding R^2^ values. Insets provide an enlarged view of the region between 1 and −2 kcal mol^−1^, highlighting the weakly interacting structures along with the corresponding R^2^ values.

As another performance measure, we compare the predicted GFN1‐xTB interaction energies directly against DFT energies in correlation plots. **Figure** **3** shows the correlation plots for Pd and Pt (all others can be found in the Supporting Information), comparing the three GFN1‐xTB parameter sets. R^2^, which quantifies the linear correlation, is indicated on each panel for all three parameter sets. In every case, the optimized parameters improve the agreement with DFT over the default set significantly. These improvements in correlation mirror the RMSE reductions discussed earlier (**Table** **3**). Not only does the absolute average error decrease, but the overall correlation relative to DFT is visibly improved, as indicated by the higher R^2^ values. The fully optimized set always yields the best performance, whereas the water‐only set bridges much of the gap between the default and optimized cases for each metal. The insets zoom into the region with small interaction energies, for which we evaluated individual R^2^ values, and reveal that in the case of the original parameterization, the data points appear to correspond to two distinct regression lines reducing the overall correlation. This is significantly improved with the optimized data sets. Obviously, the many structures at low interaction energies contribute a lot to the measured R^2^ values but have a smaller contribution to the RMSE assuming that the relative errors are equally distributed. Therefore, it is worthwhile to look at both measures to rate the improvement.

**Table 3:** Metal parameters and corresponding RMSE values. All RMSE values (kcal mol^−1^) are combined for both facets of each metal. The water‐only RMSE is calculated for optimized water parameters and default metal parameters. The final RMSE includes the metal‐specific parameter optimization. Total Sobol indices are averaged over both facets for each metal.

Metal	Parameters	Total Sobol index	Default values	Optimized values	Initial RMSE	Water‐only RMSE	Final RMSE
Ag	slater_4d levels_4d slater_5p slater_5s levels_5s levels_5p	0.16 0.13 0.08 0.07 0.03 0.01	2.720329 −9.675945 1.62 1.994885 −5.723081 −3.27343	2.763867 −11.534175 1.680464 2.393861 −6.701083 −3.928115	3.05	3.03	2.89
Au	slater_6p levels_5d shpoly_6p shpoly_5d levels_6p gam slater_5d	0.15 0.13 0.11 0.10 0.09 0.06 0.05	1.75 −10.047575 −0.051197 −0.110675 −3.296026 0.49638 3.117733	1.966673 −11.903032 −0.040957 −0.126707 −3.463758 0.595655 3.090626	3.59	3.35	2.41
Cu	levels_4p levels_3d levels_4s slater_4s slater_4p slater_3d gam3	0.18 0.16 0.13 0.11 0.10 0.09 0.07	−4.419045 −11.11405 −8.373193 1.583677 1.35 2.598287 0.023760	−4.437240 −11.388593 −9.026370 1.900412 1.358317 2.516589 0.0190081	4.14	3.65	3.34
Pd	levels_5p levels_5s gam3 slater_5p slater_5s slater_4d shpoly_4d levels_4d	0.12 0.11 0.06 0.06 0.06 0.06 0.04 0.03	−2.575 −5.724219 0.05 1.55 2.073217 2.528954 −0.232903 −12.059626	−2.115552 −6.801057 0.040 1.694208 1.770239 2.501776 −0.230124 −13.0137257	11.42	7.99	5.14
Pt	levels_6p shpoly_6p shpoly_5d gam3 levels_6s slater_6p slater_6s	0.14 0.13 0.12 0.09 0.08 0.02 0.01	−5.080419 −0.006654 −0.221693 0.109275 −7.184794 1.65 2.341428	−5.627401 −0.006432 −0.207182 0.131115 −6.046818 1.684140 2.303482	6.22	4.29	3.43

Parameter optimization can significantly enhance the predictive accuracy of semiempirical methods such as GFN1‐xTB. As we have demonstrated, by leveraging Sobol sensitivity analysis, we could systematically identify the most influential parameters, and concentrate the optimization efforts on those that matter most. Consequently, refining only the most impactful parameters in GFN1‐xTB not only boosts predictive accuracy but also lowers computational overhead, offering a simpler and more interpretable strategy for semiempirical quantum chemistry in restricted application domains.

## 4 Conclusion

We have demonstrated how a systematic optimization approach improves GFN1‐xTB predictions for water adsorption on a variety of metal surfaces. This approach ranked the parameters of the method according to Sobol sensitivity analysis. Subsequently, we carried out targeted optimizations with two schemes. In the first scheme, each metal facet (e.g., Ag(100), Cu(111)) was optimized individually, which led to significant reductions in the RMSEs. In the second scheme, which aimed at to maintain transferability to new structures in the same application domain, we refined a small set of water parameters (applicable to all surfaces) using a broad training set of 2,000 randomly selected water–metal configurations, then a similarly small number of metal parameters for each metal rather than for each facet. This general optimization scheme retained most of the accuracy gains from the facet‐specific approach while enhancing transferability across multiple surface facets and with identical optimized parameters for O and H. In both optimization schemes, the final RMSE values improved considerably over the default GFN1‐xTB parameters, particularly for challenging systems such as Pd(111) and Cu(111). A second iteration of the water parameter with optimized metal parameters confirmed the stability of the resulting parameter sets. Furthermore, adsorption curves for Ag, Au, Cu, Pd, and Pt revealed that these optimized parameters bring GFN1‐xTB predictions much closer to the DFT reference. Overall, our findings underscore the flexibility of GFN1‐xTB and demonstrate that careful adjustments to a limited subset of parameters can achieve a favorable balance between accuracy and computational efficiency. Such a domain‐specific parameterization of course depends strongly on the reference data used, and our results should not be mistaken for a criticism of the original parameterization. While we achieve major improvements for the specific systems studied, we necessarily compromise on the transferability, meaning that our suggested reparameterization scheme should be applied for applications with a clearly defined and limited scope. This, however, is not a major limitation in many cases, as our example of metal/water interfaces shows, which spans the broad fields of electrochemistry and catalysis. We therefore anticipate that such sensitivity‐based optimization schemes will find broad application in catalysis, electrochemistry, and surface science applications where reliable adsorbate–surface interactions are essential. We caution, however, that extreme care must be taken to account for all properties of interest in the objective function and include all relevant chemical species and structures in the optimization stage to avoid a restrictive lack of transferability. Future work will extend this optimization strategy to systematically refine parameters for broader classes of interfacial phenomena, including multi‐component and dynamic interfaces. Such extensions will further improve the underlying models and enhance the predictive power of GFN1‐xTB across a wider range of catalytic and electrochemical applications.

## Conflict of Interest

The authors declare no conflict of interest.

## Supporting information

Supplementary Material

## Data Availability

The data that support the findings of this study are available in the supplementary material of this article.
