# Supplementary material for: Optimizing Extended Tight‐Binding Methods for Metal‐Surface Interactions
Source: Chemphyschem. 2025 Nov 12;26(23):e202500463. doi: 10.1002/cphc.202500463 (PMC12677717; doi:10.1002/cphc.202500463)
Supplement: Supplementary file 1 — Supplementary Material [file CPHC-26-e202500463-s001.pdf]

Supporting information: Optimizing extended tight-binding methods for metal-surface interactions

Siyavash Moradi,[†] Pooria Dabbaghi,[‡] and Christopher J. Stein[*,†,¶]

†*Technical University of Munich; TUM School of Natural Sciences and Catalysis Research Center, Department of Chemistry, Lichtenbergstr. 4, 85748 Garching, Germany*

‡*Department of Energy Conversion and Storage, Technical University of Denmark, Anker Engelunds Vej 301, 2800 Kongens Lyngby, Denmark*

¶*Atomistic Modeling Center, Munich Data Science Institute, Technical University of Munich, Walther-von-Dyck Str. 10, 85748 Garching, Germany*

E-mail: christopher.stein@tum.de

1. Sensitivity plots

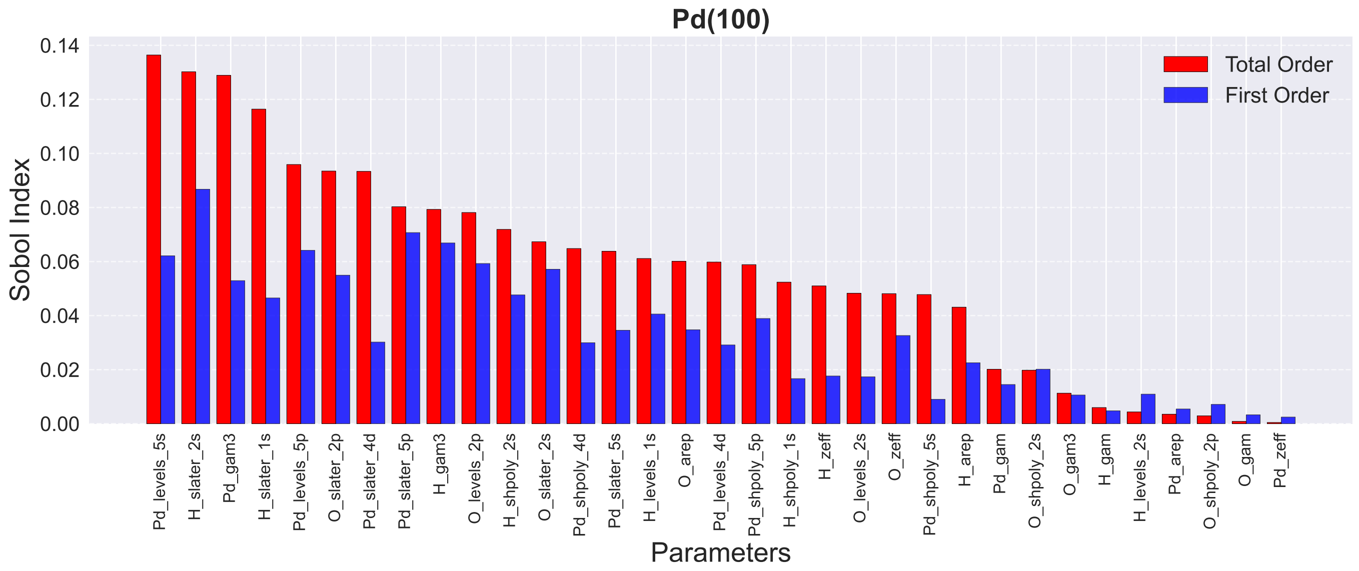

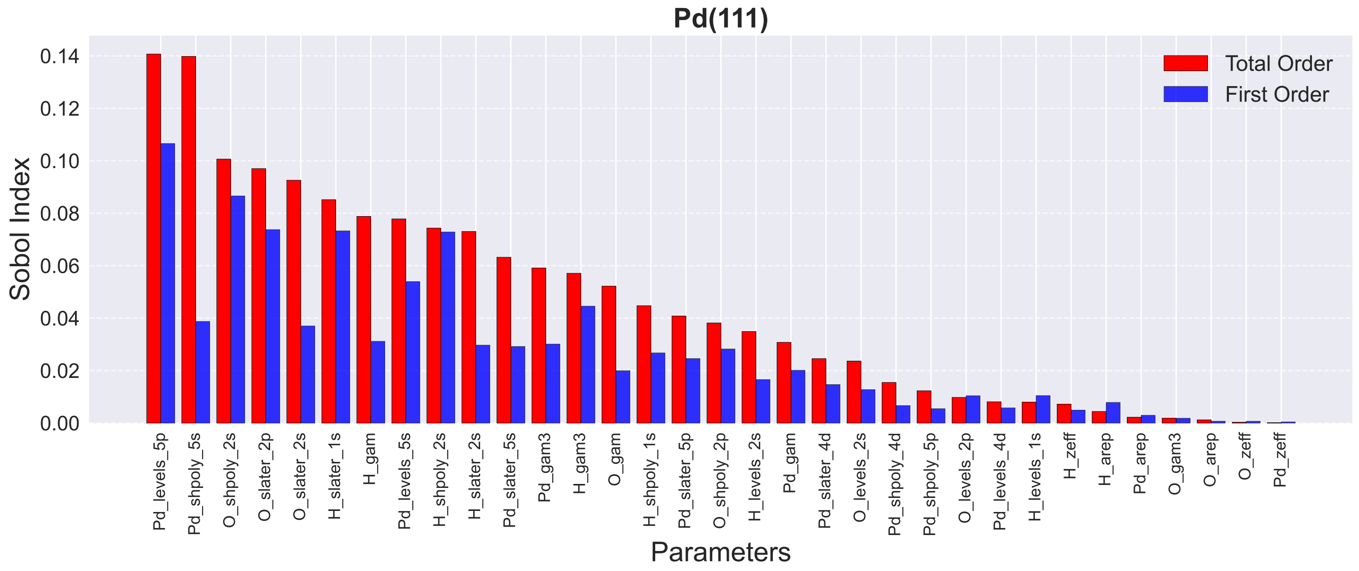

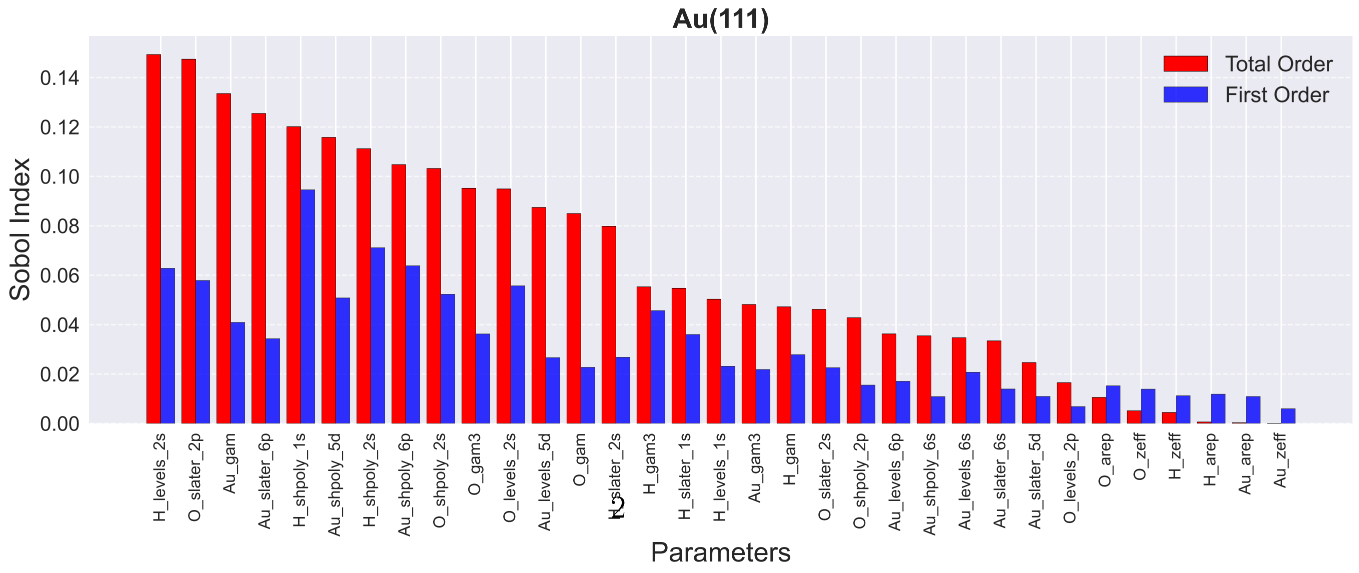

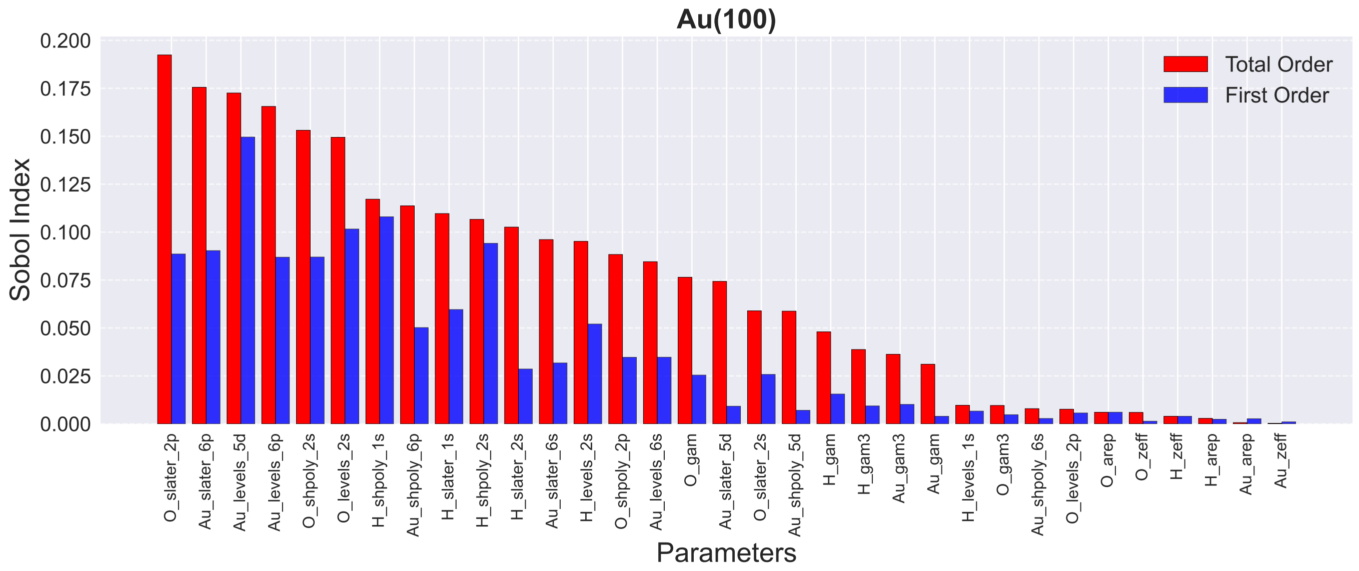
Au(100)
Sobol Index
Parameters
Total Order
First Order
0.000
0.025
0.050
0.075
0.100
0.125
0.150
0.175
0.200
O_slater_2p
Au_slater_6p
Au_levels_5d
Au_levels_6p
O_shpoly_2s
O_levels_2s
H_shpoly_1s
Au_shpoly_6p
H_slater_1s
H_shpoly_2s
H_slater_2s
Au_slater_6s
H_levels_2s
O_shpoly_2p
Au_levels_6s
O_gam
Au_slater_5d
O_slater_2s
Au_shpoly_5d
H_gam
H_gam3
Au_gam3
Au_gam
H_levels_1s
O_gam3
Au_shpoly_6s
O_levels_2p
O_arep
O_zeff
H_zeff
H_arep
Au_arep
Au_zeff

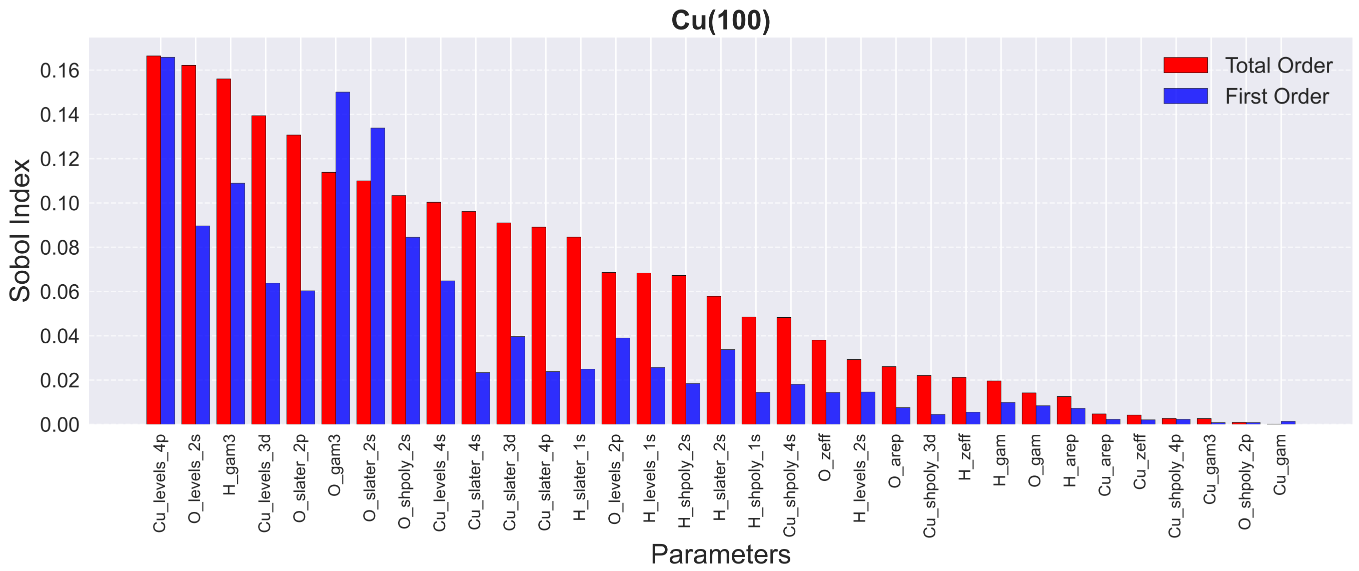
Cu(100)
Sobol Index
Parameters
Total Order
First Order
0.00
0.02
0.04
0.06
0.08
0.10
0.12
0.14
0.16
Cu_levels_4p
O_levels_2s
H_gam3
Cu_levels_3d
O_slater_2p
O_gam3
O_slater_2s
O_shpoly_2s
Cu_levels_4s
Cu_slater_4s
Cu_slater_3d
Cu_slater_4p
H_slater_1s
O_levels_2p
H_levels_1s
H_shpoly_2s
H_slater_2s
H_shpoly_1s
Cu_shpoly_4s
O_zeff
H_levels_2s
O_arep
Cu_shpoly_3d
H_zeff
H_gam
O_gam
H_arep
Cu_arep
Cu_zeff
Cu_shpoly_4p
Cu_gam3
O_shpoly_2p
Cu_gam

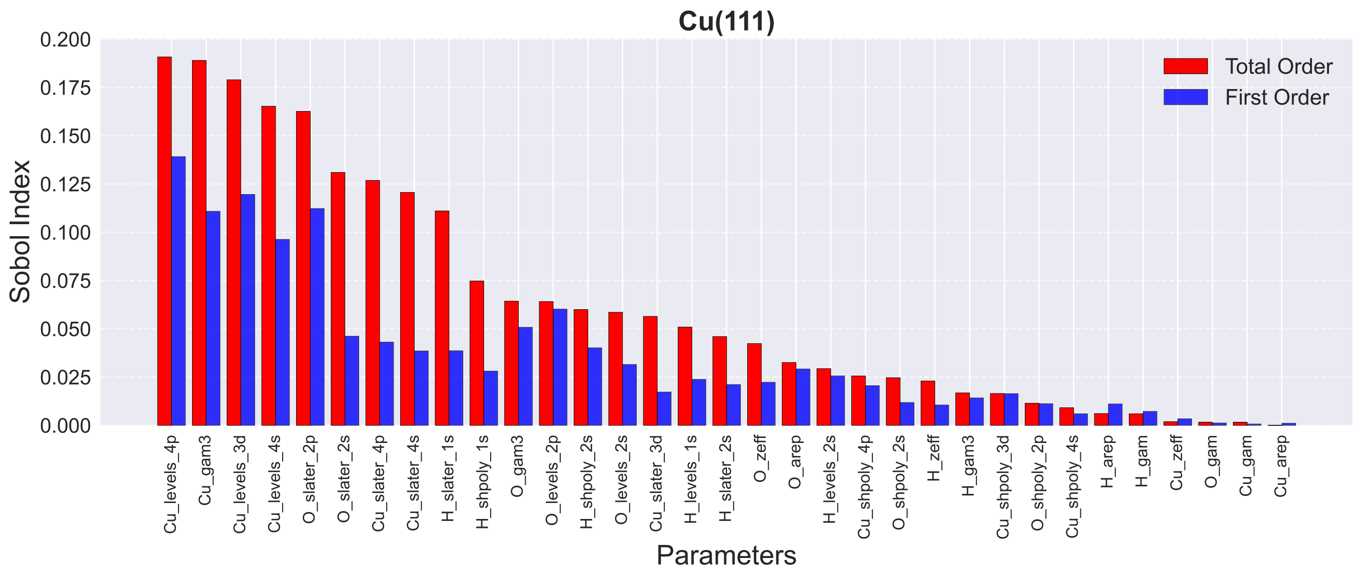
Cu(111)
Sobol Index
Parameters
Total Order
First Order
0.000
0.025
0.050
0.075
0.100
0.125
0.150
0.175
0.200
Cu_levels_4p
Cu_gam3
Cu_levels_3d
Cu_levels_4s
O_slater_2p
O_slater_2s
Cu_slater_4p
Cu_slater_4s
H_slater_1s
H_shpoly_1s
O_gam3
O_levels_2p
H_shpoly_2s
O_levels_2s
Cu_slater_3d
H_levels_1s
H_slater_2s
O_zeff
O_arep
H_levels_2s
Cu_shpoly_4p
O_shpoly_2s
H_zeff
H_gam3
Cu_shpoly_3d
O_shpoly_2p
Cu_shpoly_4s
H_arep
H_gam
Cu_zeff
O_gam
Cu_gam
Cu_arep

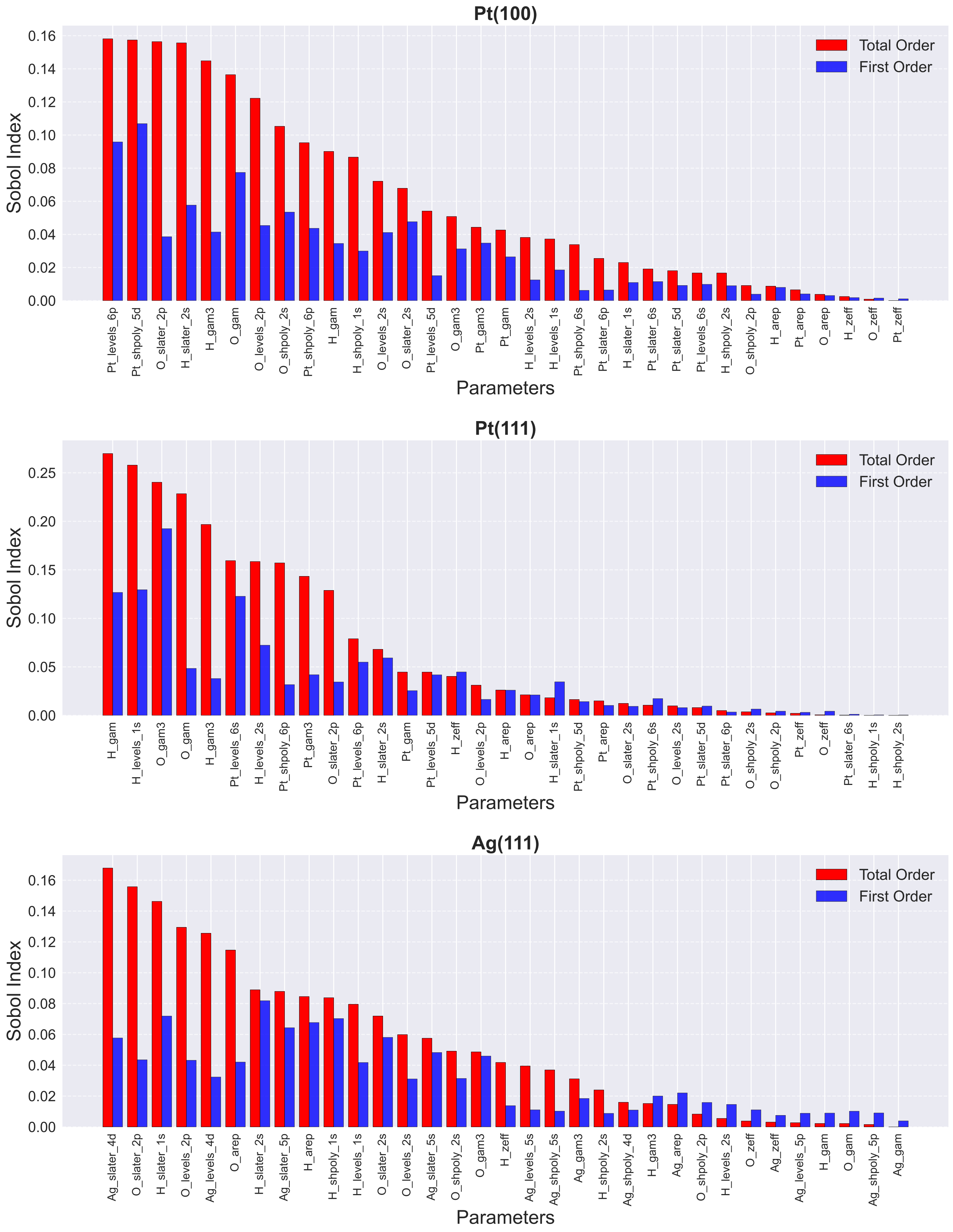
Pt(100)
Total Order
First Order
Sobol Index
0.00
0.02
0.04
0.06
0.08
0.10
0.12
0.14
0.16
Pt_levels_6p
Pt_shpoly_5d
O_slater_2p
H_slater_2s
H_gam3
O_gam
O_levels_2p
O_shpoly_2s
Pt_shpoly_6p
H_gam
H_shpoly_1s
O_levels_2s
O_slater_2s
Pt_levels_5d
O_gam3
Pt_gam3
Pt_gam
H_levels_2s
H_levels_1s
Pt_shpoly_6s
Pt_slater_6p
H_slater_1s
Pt_slater_6s
Pt_slater_5d
Pt_levels_6s
H_shpoly_2s
O_shpoly_2p
H_arep
Pt_arep
O_arep
H_zeff
O_zeff
Pt_zeff
Parameters
Pt(111)
Total Order
First Order
Sobol Index
0.00
0.05
0.10
0.15
0.20
0.25
H_gam
H_levels_1s
O_gam3
O_gam
H_gam3
Pt_levels_6s
H_levels_2s
Pt_shpoly_6p
Pt_gam3
O_slater_2p
Pt_levels_6p
H_slater_2s
Pt_gam
Pt_levels_5d
H_zeff
O_levels_2p
H_arep
O_arep
H_slater_1s
Pt_shpoly_5d
Pt_arep
O_slater_2s
Pt_shpoly_6s
O_levels_2s
Pt_slater_5d
Pt_slater_6p
O_shpoly_2s
O_shpoly_2p
Pt_zeff
O_zeff
Pt_slater_6s
H_shpoly_1s
H_shpoly_2s
Parameters
Ag(111)
Total Order
First Order
Sobol Index
0.00
0.02
0.04
0.06
0.08
0.10
0.12
0.14
0.16
Ag_slater_4d
O_slater_2p
H_slater_1s
O_levels_2p
Ag_levels_4d
O_arep
H_slater_2s
Ag_slater_5p
H_arep
H_shpoly_1s
H_levels_1s
O_slater_2s
O_levels_2s
Ag_slater_5s
O_shpoly_2s
O_gam3
H_zeff
Ag_levels_5s
Ag_shpoly_5s
Ag_gam3
H_shpoly_2s
Ag_shpoly_4d
H_gam3
Ag_arep
O_shpoly_2p
H_levels_2s
O_zeff
Ag_zeff
Ag_levels_5p
H_gam
O_gam
Ag_shpoly_5p
Ag_gam
Parameters

2. Highest errors in structure-by-structure optimization for three facets

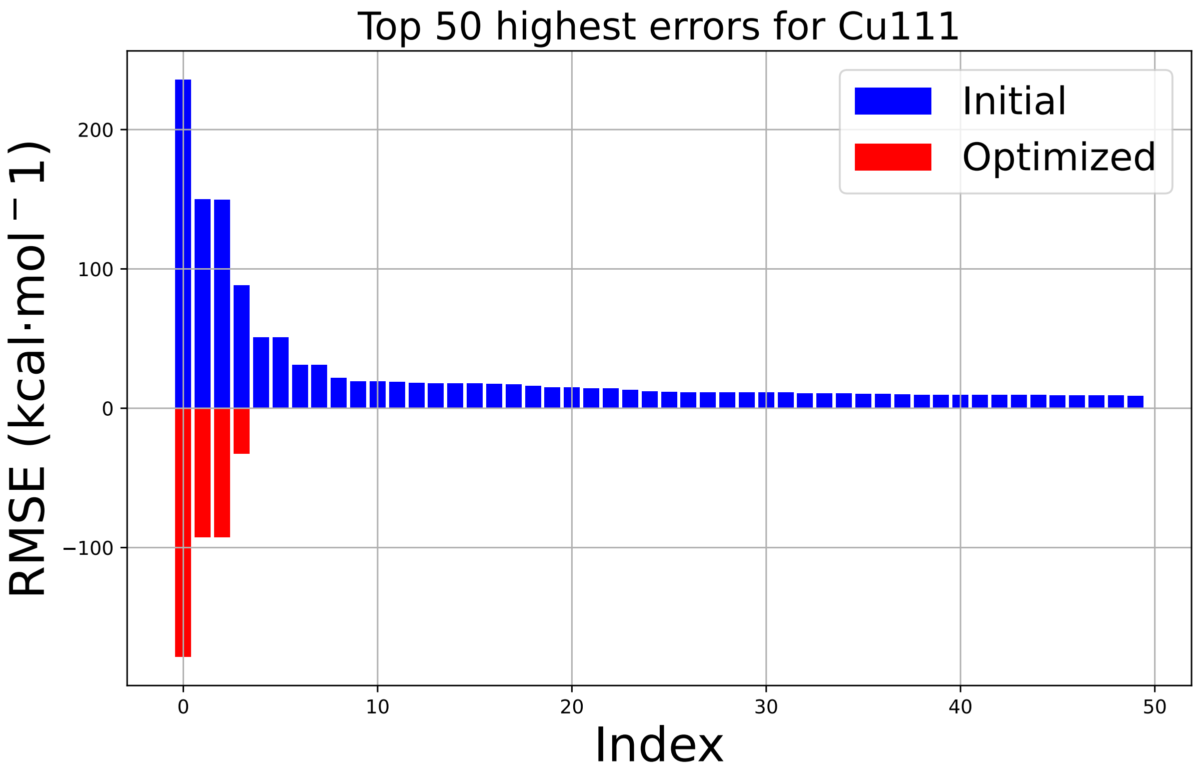

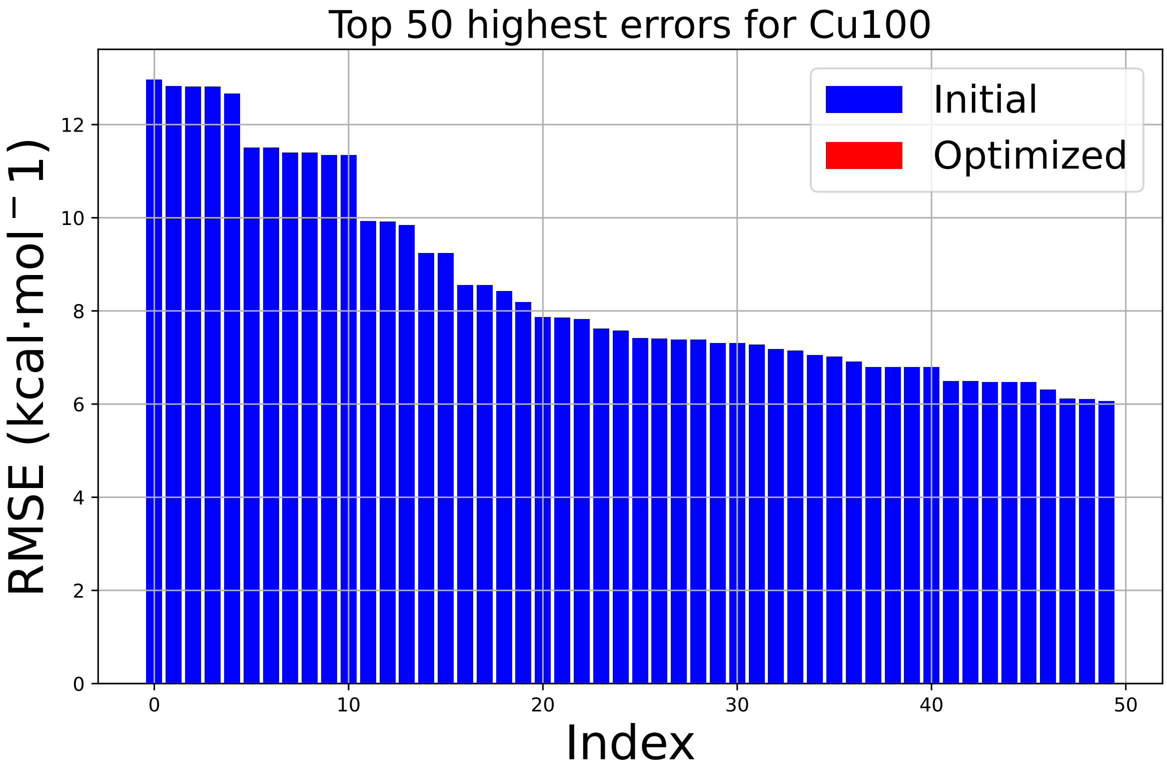
Top 50 highest errors for Cu100
Initial
Optimized
RMSE (kcal·mol⁻1)
Index
0
2
4
6
8
10
12
0
10
20
30
40
50

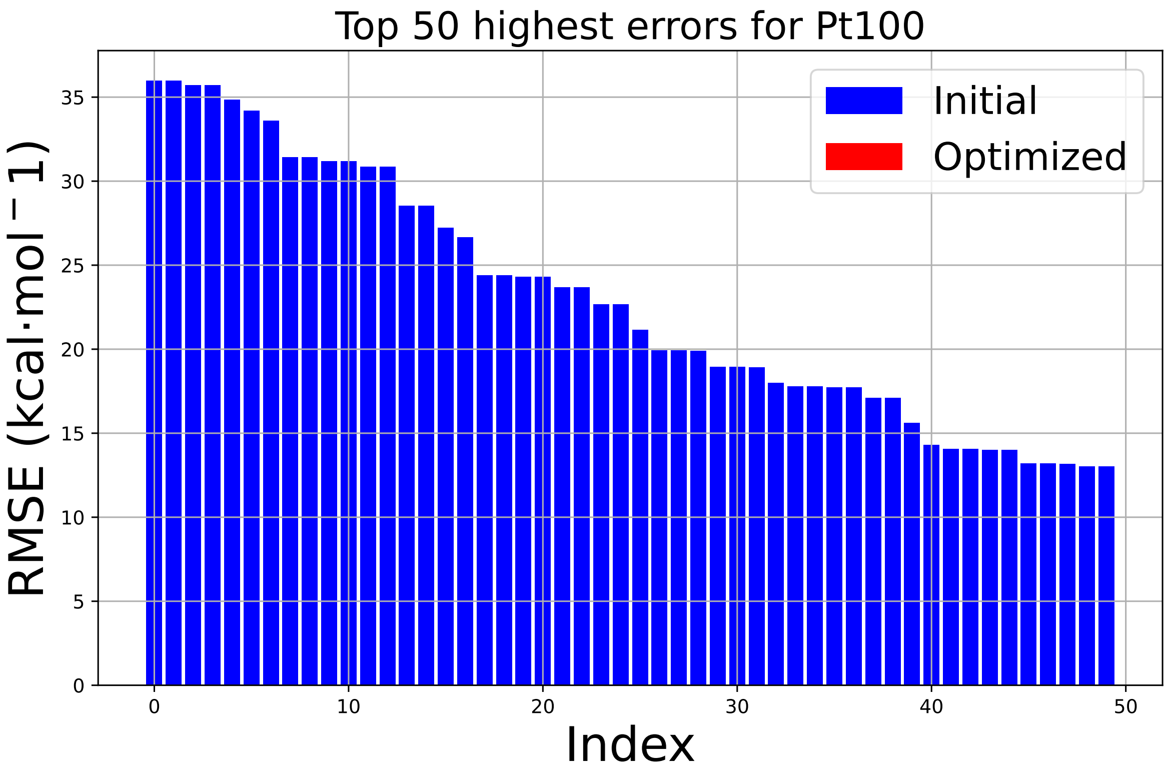
Top 50 highest errors for Pt100
Initial
Optimized
RMSE (kcal·mol⁻1)
Index
0
5
10
15
20
25
30
35
0
10
20
30
40
50

3. System specific optimized parameters

Table 1: Ag(100)

Parameter	Default	Optimized	Reversed
Ag_slater_4d	2.720329	2.612040	2.725253
O_slater_2p	2.15306	1.880140	1.91633108
O_levels_2p	-17.886554	-12.245942	-17.729217
Ag_levels_4d	-9.675945	-8.823719	-9.517482
H_slater_1s	1.20794	1.259329	1.18320806
O_arep	2.004253	2.036376	1.514800
H_slater_2s	1.993207	2.192224	2.066131
O_levels_2s	-23.398376	-28.545696	-23.891111
H_levels_1s	-10.923452	-9.489328	-7.825503
**RMSE (kcal/mol)**	3.34	1.63	1.96

Table 2: Ag(111)

Parameter	Default	Optimized	Reversed
Ag_slater_4d	2.720329	2.708482	2.750475
O_slater_2p	2.15306	1.883333	1.851547
H_slater_1s	1.20794	1.230412	1.256927
O_levels_2p	-17.886554	-11.208687	-12.549465
Ag_levels_4d	-9.675945	-11.491369	-11.579804
O_arep	2.004253	3.003495	2.305772
**RMSE (kcal/mol)**	2.81	1.92	1.99

Table 3: Au(100)

Parameter	Default	Optimized	Reversed
O_slaler_2p	2.15306	1.984588	1.711151
Au_slater_6p	1.75	2.246529	2.045354
Au_levels_5d	-10.047575	-13.831442	-12.276585
Au_levels_6p	-3.296026	-2.615905	-4.077130
O_shpoly_2s	-0.13729047	-0.18421615	-0.13248275
H_shpoly_2s	0.00	-0.08003100	0.05828319
Au_shpoly_6p	-0.05119735	-0.00480976	-0.09643833
H_slater_1s	1.20794	1.127143	1.128667
H_shpoly_2s	0.00	0.00951896	0.14490352
H_slater_2s	1.993207	2.18550449	1.97439459
**RMSE (kcal/mol)**	3.67	1.83	1.98

Table 4: Au(111)

**Parameter**	**Default**	**Optimized**	**Reversed**
H_levels_2s	-2.171902	-4.499619	-2.855522
O_slater_2p	2.15306	1.900480	2.040592
Au_gam	0.49638	1.680796	1.161535
Au_slater_6p	1.75	1.857366	1.885159
H_shpoly_1s	0.00	0.04281334	-0.00276056
Au_shpoly_5d	-0.11067532	-0.09986657	-0.08760222
H_shpoly_2s	0.00	0.04753622	0.234051788
Au_shpoly_6p	-0.05119735	-0.05479754	-0.07366816
O_shpoly_2s	-0.13729047	-0.05626431	0.161934
**RMSE (kcal/mol)**	3.51	2.16	2.40

Table 5: Cu(100)

**Parameter**	**Default**	**Optimized**	**Reversed**
Cu_levels_4p	-4.419045	-3.725715	-4.636094
O_levels_2s	-23.398376	-25.171783	-24.727928
H_gam3	0.00000000	2.727306	0.005984
Cu_levels_3d	-11.114050	-11.147343	-11.080957
O_slater_2p	2.1530600	2.133677	2.179449
O_gam3	-0.000510	-0.320523	-0.000765
O_slater_2s	2.345365	2.292113	2.181134
O_shpoly_2s	-0.137290	-0.151937	-0.205935
Cu_levels_4s	-8.3731930	-8.715220	-10.533761
**RMSE (kcal/mol)**	3.09	1.98	2.06

Table 6: Cu(111)

**Parameter**	**Default**	**Optimized**	**Reversed**
Cu_levels_4p	-4.419045	-4.990626	-4.5031797
Cu_gam3	0.0237602	0.259319	-0.14142893
Cu_levels_3d	-11.11405	-11.113973	-11.101296
Cu_levels_4s	-8.373193	-8.373193	-8.520345
O_slater_2p	2.15306	2.123605	2.158836
O_slater_2s	2.345365	2.435051	2.274351
Cu_slater_4p	1.35	1.360476	1.359379
Cu_slater_4s	1.583677	1.571212	1.46319796
H_slater_1s	1.20794	1.217939	1.17637709
**RMSE (kcal/mol)**	4.82	4.46	4.48

Table 7: Pd(100)

Parameter	Default	Optimized	Reversed
Pd_levels_5s	-5.724219	-11.724218	-6.509027
H_slater_2s	1.993207	2.014610	2.974528
Pd_gam3	0.05	-0.330825	-1.046913
H_slater_1s	1.20794	1.546145	1.713735
**RMSE (kcal/mol)**	10.42	5.57	8.24

Table 8: Pd(111)

Parameter	Default	Optimized	Reversed
Pd_levels_5p	-2.575	-2.562458	-1.41756461
Pd_shpoly_5s	0.17475085	0.1871462	0.12193466
O_shpoly_2s	-0.13729047	-0.16046528	-0.11471835
**RMSE (kcal/mol)**	12.33	10.80	11.40

Table 9: Pt(100)

Parameter	Default	Optimized	Reversed
Pt_levels_6p	-5.080419	-4.974850	-4.991936
Pt_shpoly_5d	-0.22169385	-0.16083756	-0.2241105
O_slater_2p	2.15306	2.094040	1.706953
H_slater_2s	1.993207	1.948582	1.830495
H_gam3	0.00	-0.133823	0.562765
O_gam	0.583349	0.704458	0.551358
O_levels_2p	-17.886554	-18.129430	-22.910634
O_shpoly_2s	-0.13729047	-0.17823544	-0.07746771
**RMSE (kcal/mol)**	6.82	3.02	3.93

Table 10: Pt(111)

**Parameter**	**Default**	**Optimized**	**Reversed**
H_gam	0.470099	0.431748	0.434522
H_levels_1s	-10.923452	-12.038222	-11.166807
O_gam3	-0.0005102	-0.307476	0.002227
O_gam	0.583349	0.591078	0.591165
H_gam3	0.00000000	0.232148	0.303777
Pt_levels_6s	-7.184794	-5.888089	-6.018102
H_levels_2s	-2.171902	-4.154904	-2.741859
Pt_shpoly_6p	-0.006654	0.014711	-0.006654
Pt_gam3	0.109275	0.330685	0.819048
O_slater_2p	2.15306	1.805216	1.667828
**RMSE (kcal/mol)**	4.13	2.54	2.71

5. CO adsorption plots

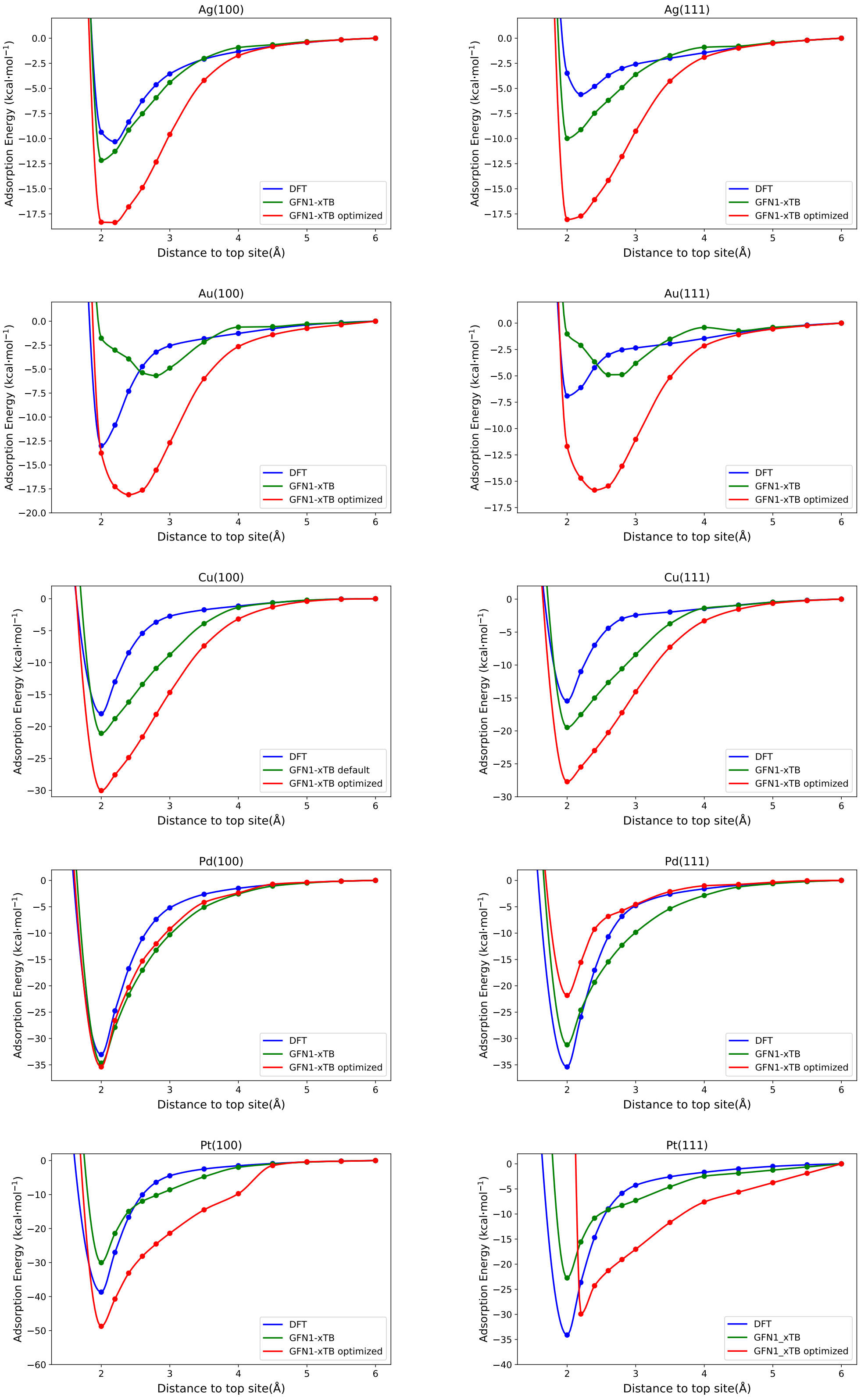

6. Further correlation plots

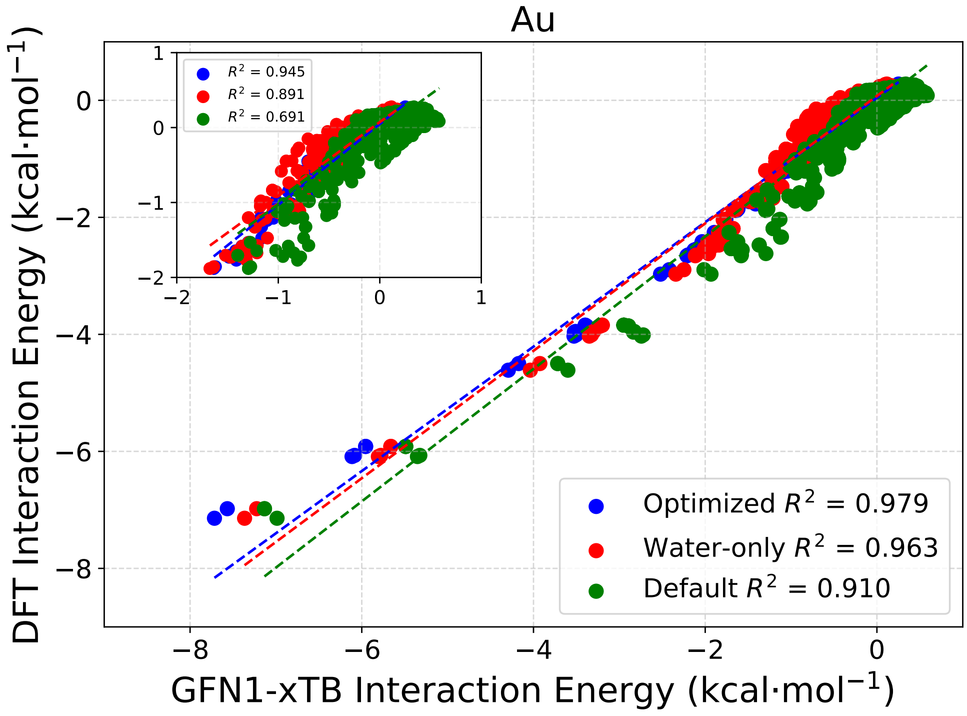

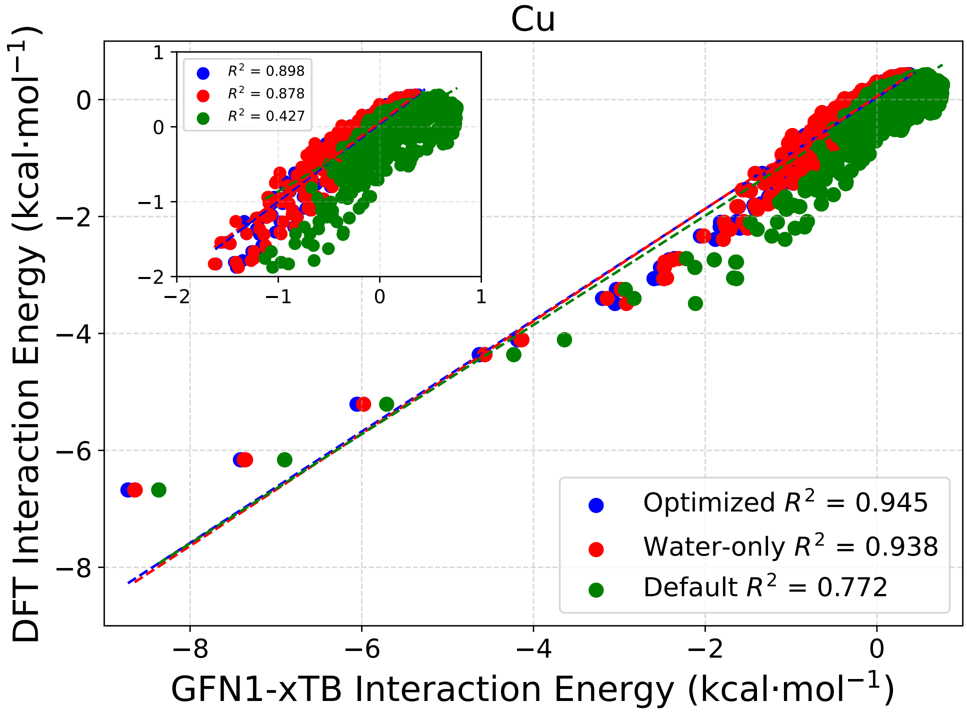

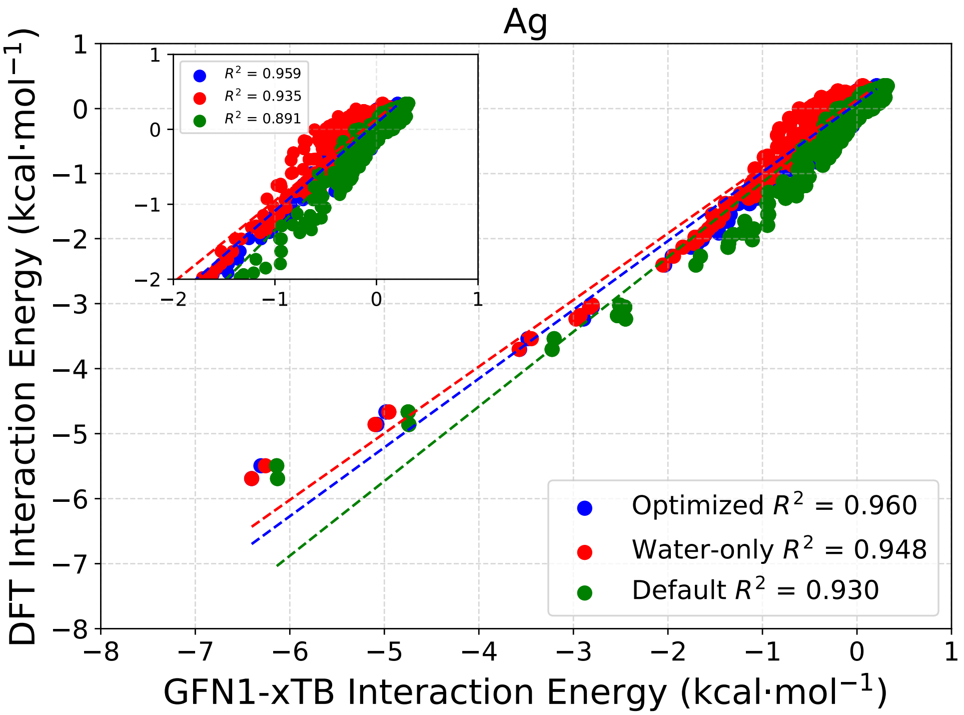
Ag
DFT Interaction Energy (kcal·mol−1)
GFN1-xTB Interaction Energy (kcal·mol−1)
Optimized R2 = 0.960
Water-only R2 = 0.948
Default R2 = 0.930
R2 = 0.959
R2 = 0.935
R2 = 0.891

7. H-up/H-down structures for transferability check

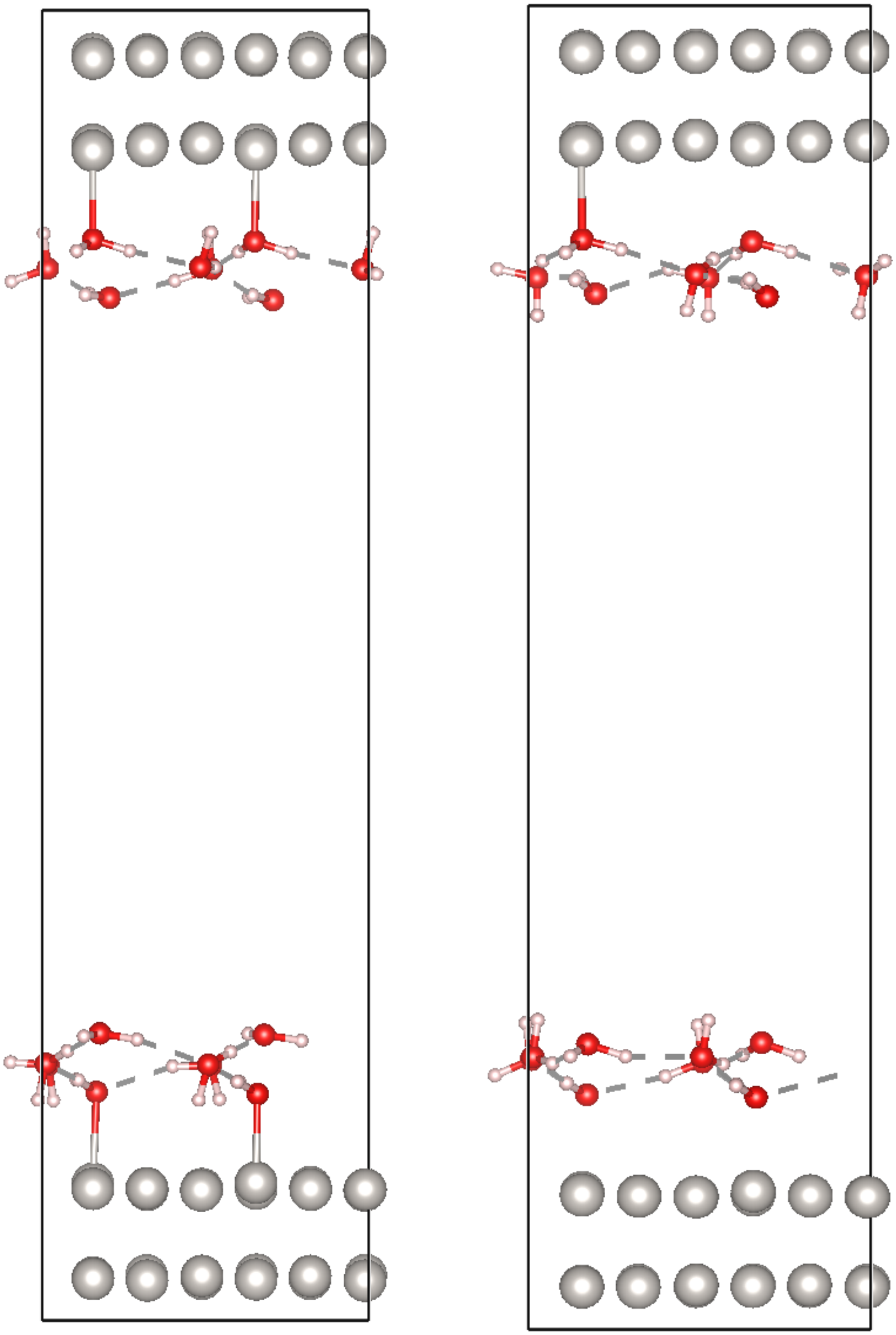

8. K_{AB} parameter

In GFN1-xTB the two-center ($\mu \in$ A, $\nu \in$ B) matrix element of the reference Hamiltonian H_0 is given by

$$\langle \phi_\mu | H_0 | \phi_\nu \rangle = K_{AB}\, \frac{1}{2}\big(k_\ell + k_{\ell'}\big)\, \frac{1}{2}\big(h_A^\ell + h_B^{\ell'}\big)\, S_{\mu\nu}\, \big(1 + k_{\mathrm{EN}}\, \Delta \mathrm{EN}_{AB}^2\big)\, \Pi\big(R_{AB}, \ell\ell'\big) \quad (\mu \in \ell(A),\ \nu \in \ell'(B)),$$

where $S_{\mu\nu}$ is the overlap between atomic orbitals ϕ_μ and ϕ_ν, h_A^ℓ and $h_B^{\ell'}$ are shell-specific on-site energies for atom A (shell ℓ) and atom B (shell ℓ'), k_ℓ and $k_{\ell'}$ scale the kinetic-energy contribution of the two shells, $k_{\mathrm{EN}}\, \Delta \mathrm{EN}_{AB}^2$ is the electronegativity-dependent correction, $\Pi(R_{AB}, \ell\ell')$ is the short-range damping function, and K_{AB} is a dimensionless element-pair factor that uniformly rescales the entire two-center coupling. The GFN1-xTB publication recommends adjusting this parameter to customize the Hamiltonian. In the standard GFN1-xTB parameterization, there is no K_{AB} parameter for an oxygen-metal pair — the parameter defaults to 1.0 — so we introduced an explicit parameter $K_{O\text{-}M}$ for four oxygen–metal pairs (Au(100), Au(111), Pt(100), and Pt(111).) and varied it over a range of values to observe its effect. In every case, the lowest RMSE was obtained when $K_{O\text{-}M}$ remained at 1.0.

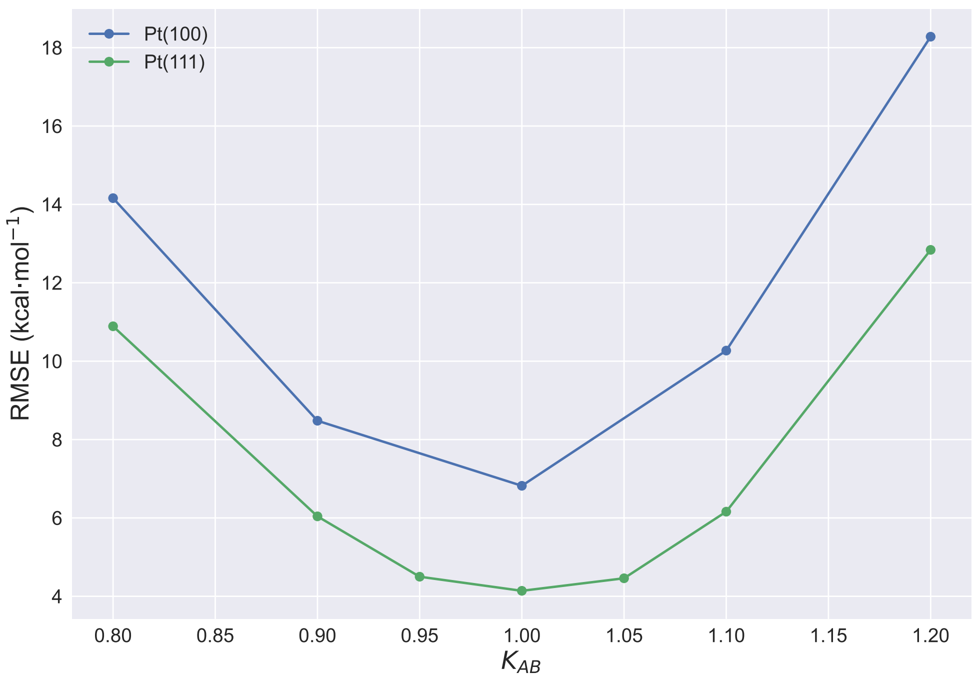
Pt(100)
Pt(111)
RMSE (kcal·mol⁻¹)
K_{AB}
18
16
14
12
10
8
6
4
0.80
0.85
0.90
0.95
1.00
1.05
1.10
1.15
1.20

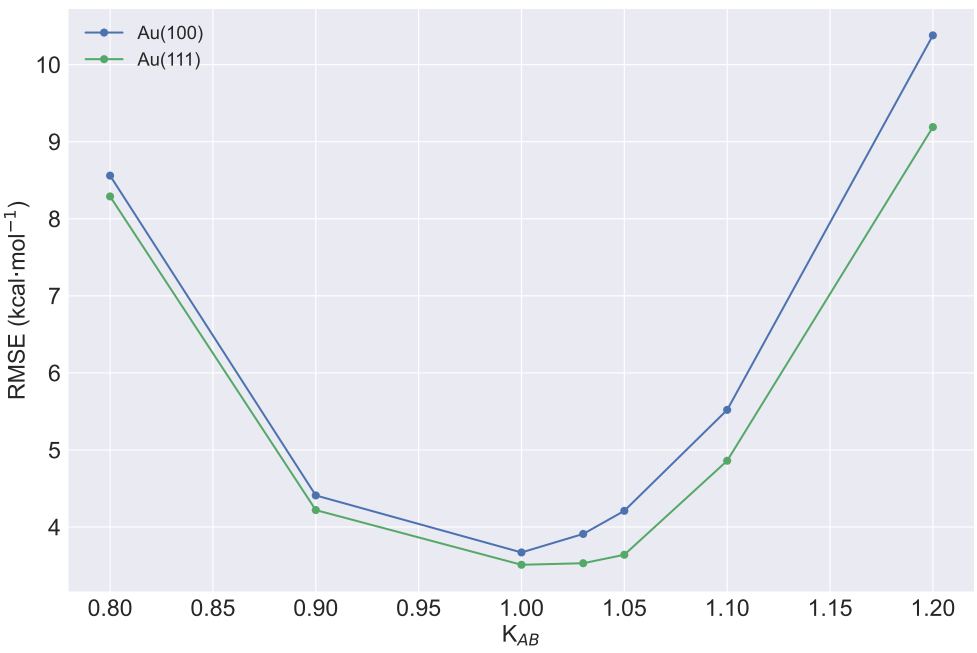
Au(100)
Au(111)
RMSE (kcal·mol⁻¹)
K_{AB}
10
9
8
7
6
5
4
0.80
0.85
0.90
0.95
1.00
1.05
1.10
1.15
1.20
